# Frequency of Blood Transfusion in Elective Coronary Artery Bypass Grafting and Its Correlation With Acute Kidney Injury at a Tertiary Care Hospital in a Lower Middle-Income Country

**DOI:** 10.7759/cureus.15184

**Published:** 2021-05-22

**Authors:** Muhammad Suleman, Khalid Samad, Hameed Ullah

**Affiliations:** 1 Anesthesiology, National Medical Centre, Karachi, PAK; 2 Anesthesia and Critical Care, Aga Khan University Hospital, Karachi, PAK; 3 Anesthesia and Critical Care, Aga Khan Health Service, Karachi, PAK; 4 Anesthesia, Aga Khan University Hospital, Karachi, PAK

**Keywords:** cabg surgery, blood transfusion, anemia, aki, acute kidney injury

## Abstract

Background

Blood transfusion remains a significant management aspect in various cardiac surgical procedures including coronary artery bypass grafting (CABG). These patients have a reportedly high incidence of transfusion of blood products. It varies considerably amongst different institutions; most variability being seen in the transfusion trigger. This case series enumerate the frequency of administration of blood products in patients during elective CABG and its association with acute kidney injury (AKI) and ascertain whether blood transfusion is justifiable at a tertiary care hospital.

Materials and methods

Using non-probability consecutive sampling, 172 patients were enrolled in the study. Patient’s preoperative hemoglobin and creatinine, intraoperative hemoglobin, transfusion trigger, number of transfusions (whole blood and packed RBCs-PRBCs), postoperative hemoglobin and creatinine in the ICU and number of transfusions in the first 24 hours were recorded.

Results

Out of the 172 patients, 96 (55.81%) patients received blood transfusion and 73 (42.69%) patients suffered from AKI. 45 (61.64%) patients with AKI received transfusion whereas 28 (38.36%) patients had no transfusion. Of these 96 patients, 45 (46.8%) received transfusion intra-operatively, 24 (25%) patients received both intra-operatively and post-operatively while 27 (28.2%) patients were transfused postoperatively. Majority of the patients, 46 (88.3%), received unjustifiable transfusion (Hb >8 g/dl) during the first 24 hours post-operatively.

Conclusion

There is marked divergence in the peri-operative use of blood products that remain on a loco-regional as well as international basis. A standardized and a multidisciplinary strategy as well as robust institutional regulation would significantly reduce inappropriate patient exposure to blood products.

## Introduction

Amongst different procedures to treat coronary artery diseases, coronary artery bypass grafting (CABG) is affiliated with significant blood loss and blood transfusion remains a crucial aspect of management in patients undergoing CABG [1,2]. Despite advances in techniques and CPB (cardiopulmonary bypass) management, the transfusion requirement is still high and varies from center to center. The frequency of transfusion in patients requiring CABG is reported between 16% and 100% [1]. Blood component administration has been identified as one of the most paramount factors associated with morbidity and mortality after CABG [3,4]. Although it can be lifesaving, it is associated with its own risks and complications including an increased risk of infections such as Hepatitis B, Hepatitis C, HIV, West Nile Virus, HTLV, etc. due to less frequent testing of the blood being transfused in resource-limited countries, adverse reactions such as acute hemolytic reaction through ABO incompatibility due to clerical errors, hypersensitivity reactions, etc., and mortality as well as prolonged hospitalization and a financial burden (high cost of testing of the blood being transfused to check for transmissible disease) in lieu of increased hospital cost.

The awareness that transfusions are not as safe as once thought they were has led to the emergence of methods and techniques to avoid them [5]. The minimum hemoglobin for transfusion, that is, the transfusion trigger has been significantly lowered. Blood conservation strategies (pharmacological and mechanical) have cost implications, therefore, identification of such population and utilization should be aimed at patients who fall in the category of high risk, which in turn will reduce the number of transfusions, proving their cost-effectiveness.

In cardiac surgery, blood transfusion rates vary between 37% and 80% [1,3,4,5]. Numerous investigators have looked into the frequency of blood transfusions in cardiac surgery [6,7] but no local data is available. It has also been observed that in addition to high rates of transfusion in cardiac surgery, transfusion practices vary considerably from institution to institution. As the above-mentioned studies show variation, this provided us a strong rationale for this study to look at the frequency of blood transfusion in patients during elective CABG at a tertiary care hospital and to compare it with the frequency of the internationally reported studies. This study might also help in developing guidelines for blood transfusion in patients planned to undergo elective open-heart surgery.

CABG is generally associated with a high incidence of post-operative acute kidney injury (AKI) leading to increased length of intensive care and hospital stay, dependence on hemodialysis and increased mortality. Some of the known risk factors are longer duration of surgery, time on the cardiopulmonary bypass machine and aortic cross-clamp time. The relationship between blood transfusion and AKI is not clear. Previously it has been suggested that the stored blood loses its morphological and biochemical integrity and can lead to impaired tissue oxygenation, increased oxidation stress and a proinflammatory reaction, leading to AKI. However, no concrete evidence exists regarding the relationship between transfusion and AKI.

## Materials and methods

After approval from the hospital ethics committee, this descriptive case series was carried out in the operating rooms and cardiac intensive care unit at a tertiary care hospital in Karachi, Pakistan for a period of six months. Using non-probability consecutive sampling technique, the sample size calculation was based on the study by Elsayed M Elmistekawy [8], which reported that the packed red blood cells transfusion (PRBC) was used in 71 patients (67.6%). On the basis of the above-mentioned study statistics, 172 patients were needed to estimate the incidence of packed red blood cells transfusion within 7% margin of error with 95% confidence interval (p = 67.7%, d = 7% n = 172) [8].

All 172 consecutive patients planned for first-time elective CABG were enrolled in this study. Patients undergoing redo surgeries, emergency CABG, complex cardiac surgeries (CABG with valvular heart surgery) were excluded. All patients were recruited at the time of preoperative anesthesia evaluation in the clinic or in the ward. They were screened for exclusion criteria. Written informed consent was obtained from patients who agreed to partake in the study. Their demographic characters including age, gender, height, weight, BMI and ethnicity were recorded. In addition, preoperative hemoglobin and serum creatinine levels were recorded.

All the patients were managed at the discretion of primary anesthetist and primary surgeon (in accordance with hospital’s contemporary standards) concerning pre and intra-operative management and postoperative care.

Patient’s preoperative hemoglobin, creatinine levels, intraoperative hemoglobin, number of transfusions (whole blood and packed RBCs or PRBCs), postoperative hemoglobin and creatinine levels in the ICU and the number of transfusions in the first 24 hours were recorded. Total length of stay was also recorded. Primary endpoint was the number of transfusions during surgery and the first 24 hours after surgery. Data was collected by the primary investigator.

Statistical analysis

Statistical Package for the Social Sciences, version 19 (SPSS Inc., Chicago, IL) was used to perform all statistical analysis. Frequency and percentage were computed for gender, ethnicity, blood transfusions on cardiopulmonary bypass (CPB), off CPB and in CICU (first 24 hours post-operatively) and the difference in creatinine levels between preop and 24 hours post-op. Mean and standard deviation was estimated for quantitative variables like age, weight, height, BMI, preoperative hemoglobin and creatinine, intraoperative level of hemoglobin, number of transfusions and postoperative hemoglobin and creatinine. Stratification was done to control effect modification like gender, age, ethnicity, weight, height, BMI, diabetes mellitus (DM), hypertension (HTN), smoking, creatinine levels, transfusion on CPB, off CPB and in CICU (first 24 hours post-operatively). Post-stratification, chi-square test was applied to observe the effect of confounder variables on outcome, that is, blood transfusion. A p-value ≤ 0.05 was considered significant.

## Results

Following were demographic characteristics of the study patients as shown in Table 1. Patients average age in the study was 60.97 ± 9.38 years. Most of the patients were male (75.6% vs 24.4%). The most common associated comorbid was hypertension (62.8%), followed by diabetes mellitus (47.7%) and smoking (29.1%).

**Table 1:** Demographics of the patients undergoing elective coronary artery bypass grafting (CABG) surgery (n = 172).

Variables	Point estimate	(Min-max) or percentage
Age (years)	60.97 ± 9.38	41-80
Weight (kg)	79.20 ± 9.31	58-115
Height (cm)	170.32 ± 6.34	154-180
BMI (kg/m^2^)	27.33 ± 3.12	19.82-41.91
Gender		
Male	130	75.6%
Female	42	42%
Ethnicity		
Baloch	10	5.8%
Gilgiti	7	4.1%
Urdu speaking	56	32.6%
Pakhtoon	28	16.3%
Punjabi	30	17.4%
Sindhi	41	23.8%
Comorbidities		
Diabetes mellitus	82	47.7%
Hypertension	108	62.8%
Smoker	50	29.1%

One hundred seventy-two patients scheduled for first-time elective CABG were screened for the frequency of transfusion. 96 (55.81%) patients received blood transfusion while 76 (44.19%) patients were not transfused either intra-operatively or post-operatively. 45 (46.8%) patients received blood transfusion intra-operatively. 24 (25%) received transfusion both intra-operatively and post-operatively while 27 (28.2%) patients were transfused post-operatively only. Table 2 shows that the average preoperative hemoglobin value among the patients enrolled was observed to be 13.24 g/dl (±1.74). Postoperatively, on arrival to CICU, the patients had an average hemoglobin of 9.97 g/dl (±1.22).

**Table 2:** Hemoglobin and transfusion status of the patients undergoing elective coronary artery bypass grafting (CABG) surgery (n = 172). CPB: cardiopulmonary bypass. ^‡^Either on or off CPB.

Variables	Point estimate	(Min-max) or percentage
Pre-operative		
Hemoglobin (g/dl)	13.24 ± 1.74	(9.0-16.8)
Intraoperative on CPB		
Hemoglobin (g/dl)	8.93 ± 1.72	(3.1-12.8)
Transfusion	39	22.7%
Intraoperative off CPB		
Hemoglobin (g/dl)	9.26 ± 1.35	(6.5-12.5)
Transfusion	46	26.7%
Postoperative on arrival to CICU		
Hemoglobin (g/dl)	9.97 ± 1.22	(5.2-13.6)
Postoperative after 24 hours		
Hemoglobin (g/dl)	9.99 ± 1.22	(5.1-13.9)
Transfusion	51	29.7%
Transfusion status, n = 96		
Intraoperative^‡^	45	46.8%
Intra^‡^ and postoperative	24	25%
Postoperative	27	28.2%

The age, weight, height, BMI and ethnicity were comparable between the patients who received transfusion versus those who didn’t. Difference in associated comorbidities between patients with and without transfusion was not statistically significant, except for smokers, in whom blood transfusion was observed to be less frequent (p = 0.001) as shown in Table 3.

**Table 3:** Comparison of demographic of the patients with and without transfusion undergoing elective coronary artery bypass grafting (CABG) surgery (n = 172).

Variables		Transfusion, n = 96	No transfusion, n = 76	p-value	
Age (years)	Mean ± SD	62.83 ± 9.99	58.61 ± 8.02	0.003	
	≤65 years	58 (60.4%)	61 (80.3%)	0.005	
	>65 years	38 (39.6%)	51 (19.7%)		
Weight (kg)	Mean ± SD	78.77 ± 10.14	80.38 ± 8.04	0.14	
	≤70 kg	24 (25%)	10 (13.2%)	0.053	
	>70 kg	72 (75%)	66 (86.8%)		
					Height (cm)	Mean ± SD	169.13 ± 6.58	171.83 ± 5.71	0.005	
≤170 cm	48 (50%)	23 (30.3%)	0.009		
>170 cm	48 (50%)	53 (69.7%)			
				BMI (kg/m^2^)		Mean ± SD	27.40 ± 3.54	27.23 ± 2.51	0.73	
≤25	24 (25%)	12 (15.8%)	0.19		
25.1 to 30	53 (55.2%)	52 (68.4%)			
>30	19 (19.8%)	12 (15.8%)			
Gender	Male	66 (68.8%)	64 (84.2%)	0.012	
	Female	30 (31.3%)	12 (15.8%)		
Ethnicity	Baloch	7 (7.3%)	3 (3.9%)	0.65	
	Gilgiti	4 (4.2%)	3 (3.9%)		
	Urdu speaking	32 (33.3%)	24 (61.6%)		
	Pakhtoon	12 (12.5%)	16 (21.1%)		
	Panjabi	16 (16.7%)	14 (18.4%)		
	Sindhi	25 (26%)	16 (21.1%)		
Comorbid	Diabetes mellitus	48 (50%)	34 (44.7%)	0.49	
	Hypertension	63 (65.6%)	45 (59.2%)	0.38	
	Smoker	18 (18.8%)	32 (42.1%)	0.001	

Out of 39 patients that were transfused on CPB, 27 (69.2%) patients were transfused with a hemoglobin level of less than 7 g/dl and 12 (30.8%) patients were transfused with a hemoglobin level of more than 7 g/dl. Forty-six patients were transfused intra-operatively after coming off from CPB, 36 (78.2%) patients received transfusion at a hemoglobin level of less than 8 g/dl and 10 (21.8%) patients received transfusion at a hemoglobin level of more than 8 g/dl. Fifty-one patients received transfusion post-operatively during the first 24 hours, 6 (11.7%) patients received transfusions at hemoglobin level of less than 8 g/dl and 46 (88.3%) patients received transfusion at hemoglobin levels of more than 8 g/dl.

Table 4 shows the frequency of AKI (defined by Kidney Disease Improving Global Outcomes (KDIGO) as an increase in serum Creatinine by ≥ 0.3 mg/dL within 48 hours or an increase of 1.5 to 1.9 times baseline which is known or presumed to have occurred within the prior seven days). 73 (42.69%) patients suffered AKI. 45 (61.64%) patients with AKI received transfusion whereas 28 (38.36%) patients had no transfusion. The frequency of AKI amongst the population requiring transfusion was not statistically significant. 57 (72.6%) of the patients having AKI were male whereas 16 (27.3%) were female. 31 (42.46%) patients with DM had perioperative AKI whereas 48 (65.75%) patients with a history of HTN developed AKI. 17 (23.28%) patients who suffered AKI were smokers.

**Table 4:** Frequency of acute kidney injury (AKI) and its correlation with blood transfusion.

		Transfusion		Total	p-value
		No	Yes		
AKI	Yes	11	22	33	0.153
		33.3%	66.7%	100.0%	
	No	65	73	138	
		47.1%	52.9%	100.0%	

Table 5 demonstrates the relationship between blood transfusion, AKI and increased length of stay (LOS), defined as a stay in the hospital for more than seven days. 30 (41%) patients suffering from perioperative AKI had LOS for more than seven days, out of these 21 patients received perioperative transfusion. 43 (59%) patients had LOS less than seven days.

**Table 5:** Relationship between blood transfusion, AKI and increased length of stay (LOS). AKI: acute kidney injury; LOS: length of stay.

LOS (days)			Transfusion		Total	p-value
			No	Yes		
>7 days	AKI	Yes	4	9	13	0.649
			30.8%	69.2%	100.0%	
		No	9	28	37	
			24.3%	75.7%	100.0%	
<=7 days	AKI	Yes	7	13	20	0.094
			35.0%	65.0%	100.0%	
		No	56	45	101	
			55.4%	44.6%	100.0%	

## Discussion

In this ever-evolving era of modern medicine, transfusion of blood products is considered the cornerstone for the management of acute blood loss and hemodynamic instability resulting from acute and massive hemorrhage. Although it is lifesaving, still even with all the sophisticated and advanced screening and nucleic acid testing of blood products which has minimized the hazards of transfusion-related infections, carry a risk of transfusion-related adverse reactions. Increase in cost, resource utilization, morbidity and mortality are observed in patients who receive transfusion during cardiac surgery. Apart from health safety, arrangement of blood products and transfusions also pose a major strain on the resources as well as a financial burden for the patient. CABG is the first line of therapy for diffuse coronary artery disease and remains the area which still requires a high number of transfusions. Transfusion practices vary considerably between different regions of the world. Variability has been noted among different centers of the same region and is considered to be multifactorial. An observational study conducted at 798 centers in the United States found transfusion frequency ranging from 7.8% to 92.8% for RBCs, 0% to 97.5% for fresh-frozen plasma, and 0.4% to 90.4% for platelets. Another prospective study carried out in Albania observed 79.87% of patients receiving blood transfusions [1]. A similar prospective study carried out in Toronto had 67.6% patients requiring a blood transfusion [8]. A retrospective review carried out in our region including elective CABG cases taking place from 2004 to 2006 had a frequency of 37.1%. The objective of our research was to discern the frequency of blood transfusion in patients who underwent elective CABG during preoperative, intraoperative and first 24 hours postoperatively and its correlation with Acute Kidney Injury (AKI). 55.81% of patients required transfusion of blood products undergoing first-time elective CABG and 42.69% patients had AKI. 61.6% of the patients who developed AKI had a history of perioperative transfusion whereas 38.4% of the patients with AKI had no transfusion, which makes transfusion of blood products as a significant risk factor in the development of AKI in our study. AKI is very common in cardiac surgeries being done on CPB (up to 30%) and blood transfusion is considered an independent risk factor for developing AKI [2]. Our results are comparable to the data available from different regions. Certain demographic features were observed and it was found that females, age < 65, weight >70 kg, BMI 25.1-30 kg/m^2^, non-smokers, coexisting DM and HTN required more blood transfusion. A retrospective review of 253 patients had similar results regarding the high frequency of transfusion among female patients as observed in our study. There’s a 2.5 times added risk of transfusion in a diabetic person undergoing CABG, as shown by a retrospective review of 700 patients undergoing CABG showed a 2.5 fold increased risk of transfusion in patients, which is comparable to our results [2,3]. A prospective study of 2,587 patients observed that smokers are less likely to receive blood transfusion during CABG when compared to non-smokers. We observed similar results in our study. The highest number of transfusions were done post-operatively (29.7%) during the first 24 hours. Various studies have attempted to discern the predictors of blood transfusions for patients undergoing CABG and have found numerous correlations between patient’s characteristics, intraoperative technique, length of bypass time, post-operative markers that can predict the need of blood product transfusions [3,4,7-9]. This area of research can be explored in our population and a predictive model identifying the high-risk patients requiring blood transfusions can be identified so that resource utilization can be optimized and patients can be spared from the additional financial burden of unnecessary transfusion of blood products. Numerous strategies have been implemented to minimize the transfusion of blood products and a consensus agreement suggests a multimodal approach for conservation of blood during surgery which includes pharmacological methods, normovolemic haemodilution and autologous transfusion and use of cell saver, etc. As mentioned earlier, transfusion of blood products is not just based on a reading of hemoglobin levels but includes hemodynamic parameters as well as the physician’s preferences. Transfusion guidelines have been present and in a continuous process of re-evaluation and assessment based mostly on the clinical data being provided. We observed that majority of the patients received unnecessary transfusion (Hb >8 g/dl) in the CICU during the first 24 hours postoperatively, necessitating the need for further studies and implication of a guideline that is to be followed. Despite having transfusion guidelines [10], it’s mostly dependent on the clinical scenario at hand, local hospital policies and the preference of the physician so as to individualize transfusion requirement for every patient as risks of transfusions need to be weighed against the benefits. Further exploration of individualizing blood transfusion can help us in developing a guideline that best suits our setting, demographic, geographical as well as lifestyle variation and to lessen the socioeconomic impact associated with it.

## Conclusions

In conclusion, there is a marked discrepancy in the peri-operative use of packed red cells/whole blood which is a result of different practices that exist nationally as well as internationally, most likely reflecting overzealous use of these products in patients who undergo cardiac procedures. These blood products should be utilized appropriately, most precisely to be based on an established transfusion guideline, thereby protecting the patient from the deleterious effects of unnecessary transfusions, scarce availability and the high cost associated with it. Local/hospital practice guidelines as well as transfusion algorithms should be continually evaluated and improved upon, a step necessary in minimizing the injudicious use of blood products, which in turn will help standardize transfusion strategies and a reduction in the exposure of cardiac surgical patients to the blood products can be achieved along with their potential exposure to AKI.
